# FOXF2 acts as a crucial molecule in tumours and embryonic development

**DOI:** 10.1038/s41419-020-2604-z

**Published:** 2020-06-05

**Authors:** Weihan He, Yuanbo Kang, Wei Zhu, Bolun Zhou, Xingjun Jiang, Caiping Ren, Weihua Guo

**Affiliations:** 1 Cancer Research Institute, Department of Neurosurgery, Xiangya Hospital, Central South University, 87 Xiangya Road, Kaifu District, Changsha, 410008 China; 2 Cancer Research Institute, Collaborative Innovation Center for Cancer Medicine, School of Basic Medical Science, Central South University, Changsha, Hunan China; 3 The NHC Key Laboratory of Carcinogenesis and The Key Laboratory of Carcinogenesis and Cancer Invasion of the Chinese Ministry of Education, Xiangya Hospital, Central South University, Changsha, Hunan 410008 China

**Keywords:** Cancer, Developmental biology

## Abstract

As a key member of the forkhead box transcription factors, forkhead box F2 (FOXF2) serves as a transcriptional regulator and regulates downstream gene expression in embryonic development, metabolism and in some common diseases, such as stroke and gastroparesis. Recent studies have shown that aberrant expression of FOXF2 is associated with a variety of tumorigenic processes, such as proliferation, invasion and metastasis. The role of FOXF2 in the development of many different organs has been confirmed by studies and has been speculated about in case reports. We focus on the mechanisms and signal pathways of tumour development initiated by aberrant expression of FOXF2, and we summarize the diseases and signal pathways caused by aberrant expression of FOXF2 in embryogenesis. This article highlights the differences in the role of FOXF2 in different tumours and demonstrates that multiple factors can regulate FOXF2 levels. In addition, FOXF2 is considered a biomarker for the diagnosis or prognosis of various tumours. Therefore, regulating the level of FOXF2 is an ideal treatment for tumours. FOXF2 could also affect the expression of some organ-specific genes to modulate organogenesis and could serve as a biomarker for specific differentiated cells. Finally, we present prospects for the continued research focus of FOXF2.

## Facts

FOXF2 exhibits inhibitory effects in most tumours.

In lung cancer (except for non-small cell lung cancer) and rhabdomyosarcoma in mice, FOXF2 mainly shows a promotive effect.

FOXF2 can both inhibit and promote HCC and breast cancer.

Regulating the expression level of FOXF2 is an ideal treatment for tumours.

Mutations in or deletions of FOXF2 occur in some patients with craniofacial deformities.

Mice with suppressed FOXF2 expression show defective organogenesis, such as aglossia, eye deformity, cleft palate and intracranial haemorrhage.

## Open questions

Does FOXF2 have a common mechanism in tumours?

What signalling pathways and gene expression does FOXF2 affect in the development of different organs?

Are there any specific mechanisms or proteins that can be targeted in the treatment of a FOXF2-affected diseases?

## Introduction

Tumours are a group of diseases characterized by abnormal cell proliferation that often forms local masses in the body. Tumour metastasis has become the main cause of cancer death^1^. After massive proliferation, malignant tumour cells invade the circulatory system from the primary site and migrate to other sites, where they continue to grow and form the same type of tumour, thus forming a metastasis^2^. The proliferation, invasion, migration and metastasis of tumours can be affected by many factors that are the focus of tumour treatments.

The process of human embryogenesis can be divided into the pre-embryonic period, embryonic period and foetal period. The embryonic stage is the key period for generating various organs and tissues, and it is the stage with the highest sensitivity to teratogenic factors. This process is regulated by a network of multiple genes and signalling pathways involving various protein families, such as the paired box (Pax) family and the Smad family^3,4^.

Forkhead box (FOX) proteins are a group of transcription factors characterized by the presence of a winged-helix domain (forkhead domain) that functions in binding DNA and always has nuclear localization^5,6^. These proteins play crucial roles in DNA repair, cell proliferation and differentiation, and organ development^7–9^. In the past few years, many FOX proteins, including FOXC2, FOXN3, FOXO1, FOXP3, FOXA2 and FOXM1, have been found to affect the proliferation, invasion and metastasis of various tumours by affecting the expression of key molecules in related signalling pathways^10–15^. In addition to the biological behaviour of tumour cells, members of the FOX family are also involved in the development of human organs, especially FOXD1^16^, FOXC1^17^, FOXI2^8^ and FOXF2, as described later.

As a member of the FOX family, early studies of FOXF2 focused on its structural characteristics and roles in embryonic development. However, current studies are mainly looking at its convoluted but interesting roles in tumour development.

Multiple studies have shown that FOXF2 can have roles in breast cancer^18,19^, lung cancer^20–22^, hepatocellular carcinoma (HCC)^23–25^, colorectal cancer (CRC)^26–29^, prostate cancer (PC)^30–32^, gastric cancer (GC)^33^, ovarian cancer (OC)^34^, rhabdomyosarcoma (RMS) in mice^35^ and other tumours (Table 1). However, FOXF2 can both promote and inhibit proliferation, invasion and metastasis in tumours, depending on the type or subtype of the tumour. Epithelial-mesenchymal transition (EMT) is a complex molecular and cellular process through which cancer cells can improve their invasion and migration abilities^36,37^. FOXF2 can promote EMT by downregulating the expression of E-cadherin^37^. Overexpression of β-catenin and low expression of E-cadherin are associated with a poor prognosis for nasopharyngeal carcinoma^38^. FOXF2 may promote or inhibit tumour development by affecting the EMT process, G1-S cell cycle transition^33,39^, Wnt/ beta-catenin pathway^33,40–42^, BMP/SMAD pathway^43^, and vascular endothelial growth factor-C (VEGF-C)/vascular growth factor receptor 3 (VEGFR3) pathway^44^. In addition, the expression of FOXF2 can be downregulated by a variety of microRNAs. During embryonic development, FOXF2 expression is influenced by SHH signalling, GLI, etc., which affects the levels of various proteins paramount for the development of specific organs, such as platelet-derived growth factor receptor β (Pdgfrβ), wingless-type MMTV integration site family member 5a (Wnt5a), and surfactant proteins A (SPA)^4,45–48^. The aberrant expression of FOXF2 in developing organs causes various congenital malformations, such as cleft palate, persistent hyperplastic primary vitreous, and atrioventricular septal defect^45,49,50^.

**Table 1:** A summary of FOXF2 expression, mechanism, function in tumours.

Tumour	Expression	Mechanism	Function	Reference
Breast cancer (in mice)	–	TGF-β/↑FOXF2/↓E-cadherin, ↑Zeb1, ↑Zeb2, ↑Noxa, ↓Id2, ↓miR-200 family, ↓betacellulin, ↓amphiregulin/↑EMT	Promoting apoptosis, migration	^37^
Breast cancer	–(BLBC)	1. MAZ/↑FOXF2/↓Twist1/EMT↓ 2. MAZ/↑FOXF2/-	1. Inhibiting migration 2. Promoting proliferation	^61,62^
Breast cancer	Upregulation	↑FOXF2/↑BMP/ SMAD pathway, ↑BRGs	Promoting bone metastasis	^43^
Breast cancer	Upregulation	↓miR-200c/↑FOXF2/↓E-cadherin, ↑Zeb1/↑EMT	Promoting invasion, migration, metastasis	^18^
Breast Cancer	Upregulation in BLBC	↑FOXF2/↑EMT	Promoting invasion, migration	^39^
Breast cancer	Downregulation	↑miR-301/↓ FOXF2/↑Wnt5a	Inhibiting proliferation, migration	^57^
Breast cancer	Downregulation in BLBC	↓FOXF2/↑Twist1/↑ EMT	Inhibiting metastasis	^91,61^
Breast cancer	Downregulation in BLBC	↓FOXF2/↑FOXC2/↑EMT	Inhibiting proliferation, invasion, metastasis	^19^
Breast cancer	Downregulation in BLBC	↓SP1/↓FOXF2	Inhibiting proliferation	^56^
Breast cancer	Downregulation in BLBC	↓FOXF2/↑VEGFR3	Inhibiting lymphatic metastasis	^44^
Breast cancer	Downregulation in luminal-type and HER2-positive breast cancer	↓FOXF2/↑CDK2-RB-E2F cascade	Inhibiting proliferation	^39^
Lung cancer	Downregulation in NSCLC	–	Inhibiting cancer progression	^20^
Lung cancer	Upregulation	↑FOXF2/↓E-cadherin, ↓miR-200/↑EMT	Promoting, invasion, migration, metastasis	^22^
CRC	Downregulation	↑miR-130a, ↑miR-182/↓FOXF2/↑β-catenin	Inhibiting proliferation, invasion, migration	^27,28^
CRC	Downregulation	↑LSD1/↓FOXF2/↑EMT	Inhibiting proliferation, invasion, migration	^29^
HCC	Downregulation	↑miR-519a/↓FOXF2/↑E-Cadherin, ↓ vimentin/↓EMT	Inhibiting proliferation, colonization, metastasis, promoting invasion, migration	^23,25^
PC	Downregulation	↑miR-182-5p/↓FOXF2/↑MMP1, ↓TIMP3/↓ECM	Inhibiting proliferation, invasion, migration	^31,32^
ESCC	Downregulation	DNA promoter methylation/↓FOXF2	Inhibiting cancer progression	^68,69^
Cervical cancer	Downregulation	↓FOXF2/↑β-catenin/↑EMT	Inhibiting proliferation, invasion, migration	^42^
OC	downregulation	↓ADAMTS9-AS2/↑miR-182-5p/↓FOXF2/↑EMT	inhibiting proliferation, invasion, migration	^34^
GC	Downregulation	DNA promoter methylation/↓FOXF2/↓E3 ligase IRF2BPL /↑Wnt/β-catenin pathway	Inhibiting proliferation, invasion, migration	^33^
Intestinal Adenomas (in mice)	Downregulation	↓FOXF2/↓SFRP1/↑Wnt	Inhibiting formation and growth	^40,41^
RMS (in mice)	Upregulation	↑FOXF2/↓p21	Promoting proliferation	^35^

”↑” means upregulation,”↓” means downregulation; *EMT* epithelial–mesenchymal transition, *SFRP1* secreted frizzled related protein 1, *Zeb1* zinc finger E-box-binding homeobox 1, *MAZ* Myc-associated zinc finger protein, *BLBC* basal-like breast cancer, *BRGs* bone-related genes, *LSD1* lysine-specific demethylase 1, *Wnt5a* wingless-type MMTV integration site family member 5a, *TNBC* triple-negative breast cancer, *VEGFR3* vascular endothelial growth factor receptor 3, *Id2* inhibitor of differentiation 2, *NSCLC* non-small cell lung cancer, *HCC* hepatocellular carcinoma, *PC* prostate cancer, *MMP1* matrix metalloproteinase 1, *RB* retinoblastoma tumour suppressor protein, *TIMP3* tissue inhibitor of metalloproteinase 3, *ECM* extracellular matrix, *ESCC* esophageal squamous cell carcinoma, *OC* ovarian cancer, *IRF2BPL* interferon regulatory factor 2-binding protein-like, *RMS* rhabdomyosarcoma.

This review aims to be the first to systematically summarize the different roles of FOXF2 in different types or subtypes of tumours and the underlying molecular mechanisms, thus revealing the possible clinical applications of FOXF2 and demonstrating the roles of FOXF2 in embryonic development and pathogenic mechanisms. We hope that this article will provide clinical workers and researchers with a comprehensive understanding of the structure and function of FOXF2 and provide new ideas for treatment strategies for related diseases.

## The structure of FOXF2

**Fig. 1:**
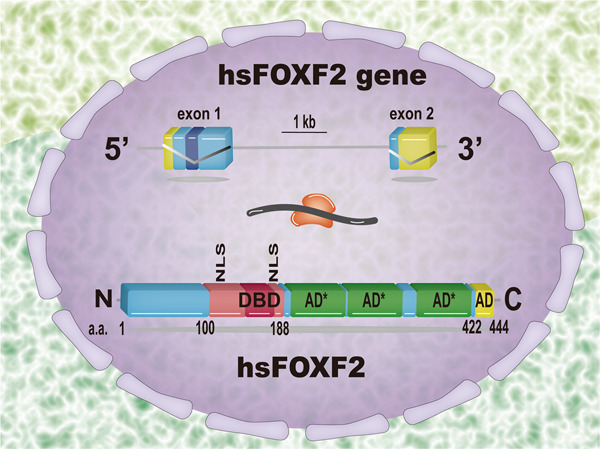
The structure of the human FOXF2 gene. Lines represent the intron and untranscribed flanking sequences, and boxes represent exons. Boxes at both ends mark untranslated sequences, other areas denote coding sequences in the human FOXF2 gene, and the dark box in exon 1 emphasizes the site of the forkhead motif. (Below) The structure and functional domains of the human FOXF2 protein are shown. DBD, DNA binding domain; NLS, nuclear localization signal; AD, activating domain; and hs, Homo sapiens. *Three synergistic subdomains comprising activating domain 2, whose exact location has not yet been revealed.

Located on chromosome 6p25.3 in humans, *FOXF2* is composed of two exons split by an intron of 3.6 kd^51^. It encodes the transcriptional regulation factor FOXF2 (formerly known as ‘FKHL6’ and ‘FREAC2’), containing 444 amino acid residues. The forkhead domain, whose C- and N-terminal domains take part in the nuclear localization, is responsible for binding cis-elements of downstream genes. In addition, the 23 amino acids at the C-terminal of the FOXF2 encoded by exon 1 act as an independent activation domain that transactivate transcription of downstream genes, which is one transcriptional activation domain, AD1. The other domain, AD2, consists of ~200 discrete amino acids in the central portion of FOXF2 (Fig. 1). Activation mediated by AD2 depends on the tertiary structure of FOXF2. However, there is no synergistic effect between AD1 and AD2^6^.

## The role of FOXF2 in tumours

### General description

**Fig. 2:**
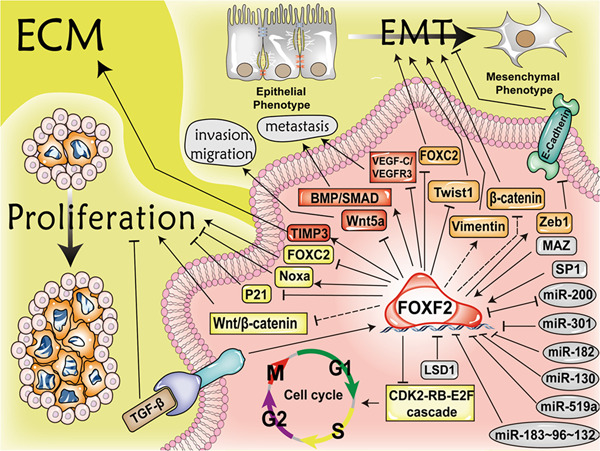
**Overall actions of FOXF2 in tumours**. In mice, TGF-β can activate the expression of Noxa protein and inhibit EGF-mediated survival signal transduction by upregulating FOXF2 expression to achieve apoptosis of cancer cells. FOXF2 can be upregulated by MAZ and mediate the function of MAZ on promoting the proliferation of cancer cells. FOXF2 can also directly inhibit the expression of P21 and promote the proliferation of tumour cells in mice. FOXF2 inhibits proliferation by inhibiting the CDK2-RB-E2F cascade or the Wnt/β-catenin pathway. FOXF2 mediates the effect of SP1 to inhibit proliferation. FOXF2 can downregulate the expression of FOXC2 to inhibit the proliferation and EMT of tumour cells. FOXF2 can promote EMT by downregulating the expression of E-cadherin and miR-200 and by upregulating vimentin expression. FOXF2 can also inhibit Twist 1 or downregulate β-catenin to inhibit EMT. FOXF2 promotes tumour metastasis by activating the BMP/SMAD pathway, and it inhibits lymphatic metastasis by inhibiting the VEGF-C/VEGFR-3 pathway. To inhibit the migration of tumours, FOXF2 downregulates the expression of Wnt5a or inhibits the degradation of ECM by activating TIMP3. EMT, epithelial–mesenchymal transition; Zeb1, zinc finger E-box-binding homeobox 1; MAZ, Myc-associated zinc finger protein; LSD1, lysine-specific demethylase 1; Wnt5a, wingless-type MMTV integration site family member 5a; VEGFR3, vascular endothelial growth factor receptor 3; TIMP3, tissue inhibitor of metalloproteinase 3; and ECM, extracellular matrix.

Many studies have shown that FOXF2 plays an important role in tumours, but its role is not identical or even opposite in different tumours or different subtypes of the same tumour. This leads to different tumour treatment options based on FOXF2. FOXF2 exhibits inhibitory effects in most tumours. In lung cancer (except for non-small cell lung cancer^20,21^) and RMS in mice^35^, FOXF2 mainly shows a promotive effect. In HCC^23^ and breast cancer^18,19^, the effect of FOXF2 can be to inhibit and to promote. The roles of FOXF2 in tumours are summarized in Fig. 2.

### Different roles of FOXF2 in different tumours

#### Breast cancer

Among women, breast cancer is the most common cancer and the main cause of cancer death^52^. FOXF2 plays multiple roles in breast cancer.

FOXF2 promotes invasion, migration and metastasis of breast cancer cells. One study showed that miR-200c can inhibit the metastasis of breast cancer cells and significantly reduce FOXF2 expression^18^. Lo et al.^39^ found that FOXF2 was overexpressed in BLBC and was necessary for the migration, invasion and anchorage-independent growth of BLBC cells. In addition, research from Lo et al. on metabolic rewiring of tumours found that FOXF2 may play a role in promoting the development of basal-like triple-negative breast cancers (TNBC) by negatively regulating PLA2G12A, PMVK and other tumour-suppressive metabolic genes^53^. FOXF2 can promote bone metastasis of breast cancer^43^. The function of FOXF2 is to act as a master transcription factor and as a regulator of the epithelial-to-osteomimicry transition to make cancer cells metastasize to bone through pleiotropic transactivation of the BMP/SMAD signalling pathway and of BRGs that are expressed at early stages of bone differentiation; these changes lead to osteolytic bone lesions in bone metastasis^43^.

FOXF2 inhibits proliferation, invasion, migration, metastasis and drug resistance of breast cancer cells. The FOXF2 gene is often silenced in luminal-type and HER2-positive breast cancers^39^. Further studies showed that FOXF2 could help block the G1-S transition of the cell cycle by inhibiting the CDK2-RB-E2F cascade, thus inhibiting the development of luminal-type and HER2-positive breast cancer^39^. In addition, Feng et al^19^. found that FOXF2 can directly target FOXC2 and transcriptionally inhibit FOXC2 in BLBC cells. FOXC2-mediated EMT may be another mechanism by which cancer cells initiate and maintain drug resistance^54^. Therefore, FOXF2 can achieve anticancer effects by inhibiting FOXC2, which suppresses the invasiveness and drug resistance of BLBC cells. Additionally, SP1 can promote the proliferation of BLBC cells via direct binding to the proximal promoter region, resulting in an increase in the transcriptional activity of FOXF2; however, DNA methylation inhibits this binding^55,56^. Some microRNAs inhibit the anticancer effects of FOXF2. MiR-301 can affect lymph node metastasis of tumour cells, downregulate the expression of FOXF2 and reduce the inhibition of FOXF2 on the expression of Wnt5a^57^. Whether FOXF2 can mediate the function of MiR-301 needs further experiments to prove. In TNBC tissues and cells, miR-182 also promotes TNBC cell proliferation and migration by downregulating FOXF2^58,59^. A deficiency of FOXF2 can activate the VEGF-C/VEGFR3 signalling pathway in BLBC cells so that cancer cells have lymphangiogenic mimicry characteristics and enhanced lymphatic metastasis abilities^44^. Chen et al.^60^ found that when the Twist1 gene was deleted, tumour cells would undergo MET transformation. FOXF2 can inhibit the EMT programme of tumour cells by repressing Twist1 transcription, thereby reducing the metastatic capacity of BLBC cells^61^.

FOXF2 can play a dual role in the development of breast cancer by mediating TGF-β or MAZ. A recent study has shown that in mice, FOXF2 expression can be upregulated by TGF-β, and FOXF2 can significantly downregulate E-cadherin expression levels by promoting the expression of transcriptional repressors of E-cadherin, Zeb1 and zinc finger E-box-binding homeobox 2 (Zeb2); moreover, the expression of inhibitor of differentiation 2 (Id2) and members of the miR-200 family can be inhibited to facilitate the invasion and metastasis of breast cancer cells^37^. During TGF-β-induced EMT, FOXF2 can negatively regulate transduction of the epidermal growth factor receptor (EGFR)-mediated survival signal by activating the transcription of the proapoptotic protein Noxa and directly inhibiting the transcription of betacellulin and amphiregulin, which are ligands of EGFR; these activities achieve apoptosis mediated by TGF-β in NMuMG cells^37^. Moreover, MAZ can promote FOXF2 expression by activating the promoter of *FOXF2* in BLBC cells, thus negatively regulating Twist1 expression to inhibit EMT and metastasis of BLBC cells^61,62^. However, FOXF2 plays a key role in the proliferation of BLBC cells that is promoted by MAZ^62^. The dual function of the MAZ-FOXF2 axis reflects the multipotency of multifunctional transcription factors in regulating different stages of tumourigenesis and development, and it illustrates the complexity of diagnosis and treatment of breast cancer^62^.

Many functions of FOXF2 in breast cancer are depend on the internal environment of cells and tissues. In addition, different cell lines, experimental materials and experimental methods used by the researchers also lead to the various effects of FOXF2.

#### Lung cancer

Lung cancer is the most common cancer, accounting for 11.6% of total cases. It is also the leading cause of cancer deaths, accounting for 18.4% of the total cancer deaths^52^.

In non-small cell lung cancer (NSCLC), FOXF2 plays an anticancer role. The study by Kong et al^20^. showed that the expression of FOXF2 in NSCLC was lower than it was in normal lung tissue, and the expression of FOXF2 was positively correlated with the survival time of patients. In addition, a single-nucleotide polymorphism (SNP) FOXF2rs1711972A>C is associated with a better survival rate for surgical treatment of NSCLC^21^.

FOXF2 also has a positive effect on lung cancer. Many kinds of microRNAs can inhibit lung cancer by targeting FOXF2. The ectopic expression of the miR-183~96~182 clusters inhibits the migration and invasion of cancer cells, and the miR-200 family has a close regulatory effect on its expression. These two microRNA families both target FOXF2 and inhibit its expression^22^. The strong invasion, migration and metastasis induced by FOXF2 in lung cancer cells are closely related to Zeb1^22^. At the same time, FOXF2 can function independently of Zeb1 to inhibit the expression of E-cadherin and miR-200, which induces invasion and metastasis^22^.

Air pollution from smoking and bituminous coal combustion are the main causes of lung cancer^63,64^. A study from Wei et al^63^. confirmed that dibenz [a, h] anthracene, a carcinogen of bituminous coal combustion, can upregulate the lncRNA CAR intergenic 10 (CAR10) by increasing FOXF2 expression, and then CAR10 can bind to and stabilize the transcription factor Y-box-binding protein 1 (Yb-1), which results in the upregulation of EGFR and the proliferation of lung cancer cells. Tharappel et al^65^. found that cigarette smoke can increase the DNA binding activity of FOXF2, the mechanism and consequences of which need further examination and validation.

Geng et al^66^. revealed an increase in the expression of miR-301b and a decrease in the expression of its target gene FOXF2 in hypoxic lung cancer cells, suggesting that FOXF2 may play an important role in the hypoxia response of lung cancer cells.

#### Colorectal cancer

CRC is one of the leading causes of cancer deaths worldwide^52^, and FOXF2 predominantly plays an anticancer role in CRC. A study by Hauptman et al. showed that the FOXF2 gene was hypermethylated in 81.1% of CRC samples and was downregulated in 98.9% of the samples^26^. In addition, Chen, Zhang et al^27,28^. found that the expression of miR-130a and miR-182 in CRC was significantly upregulated, and they directly targeted FOXF2 to downregulate its expression to promote the proliferation, invasion and migration of CRC cells. In addition, Chen et al.^29^ found that FOXF2 was a target gene of lysine-specific demethylase 1 (LSD1), which can affect the proliferation, metastasis and invasion of colon cancer by downregulating FOXF2 expression.

#### Hepatocellular carcinoma

HCC composes 75–85% of all liver cancer cases^52^. In HCC, FOXF2 can inhibit the proliferation, colonization and metastasis of HCC cells but can also improve their invasion and migration ability. Dou et al^23^. found that the downregulation of FOFX2 expression in HCC cells resulted in the increase of E-cadherin and the decrease of vimentin, induced mesenchymal epithelial transformation (MET) of HCC, inhibited the invasion and migration of HCC cells, promoted the proliferation of HCC cells, enhanced the colonization of circulating HCC cells, and consequently promoted the formation of metastatic nodules. Furthermore, Shi et al^24^. silenced FOXF2 gene by treating HCC cells with an interfering RNA and found that it could significantly promote the proliferation and decrease the apoptosis of cancer cells. Moreover, Shao et al.^25^ indicated that in HCC tissues, miR-519a can enhance proliferation and inhibit apoptosis of HCC cells by downregulating FOXF2.

#### Prostate cancer

PC is the most common cancer; it has been diagnosed in 105 countries and is a considerable cause of cancer deaths worldwide^52^. In PC, FOXF2 mainly plays an anticancer role. Van der Heul-Nieuwenhuijsen et al.^30^ found that FOXF2 expression increased in the normal transitional zone of the prostate and in benign prostatic hyperplasia, but it was decreased in PC. Further studies showed that FOXF2 could regulate the ECM level by lowering matrix metalloproteinase 1 (MMP1) and increasing tissue inhibitor of metalloproteinase 3 (TIMP3) to control the balance between MMPS and TIMPS. Van der Heul-Nieuwenhuijsen et al.^31^ believed that the low incidence of PC in the transitional zone may be due to the high expression of FOXF2, resulting in a more stable environment. It has been reported that miR-182 is highly expressed in PC^67^. One study identified *FOXF2* as a target gene for miR-182-5p and found that increased expression of FOXF2 can inhibit the proliferation, migration and invasion of PC cells after miR-182-5p was knocked out^32^.

#### Esophageal squamous cell carcinoma

Esophageal squamous cell carcinoma (ESCC) is a histologic subtype of esophageal cancer (EC)^52^, and FOXF2 mainly plays an anticancer role in ESCC. Zheng et al.^68^ analysed 188 ESCC clinical samples and found that the expression of FOXF2 decreased in cancer tissues; further, a low level of FOXF2 mRNA was associated with a higher lymph node metastasis rate. Additionally, Chen et al.^69^ validated that the highly methylated *FOXF2* promoter was associated with a low survival rate in patients with ESCC.

#### Other tumours

In cervical cancer, OC, bladder cancer, GC and intestinal adenoma, FOXF2 is a cancer suppressor, while in RMS, it is a promoter. Zhang et al^42^. observed low expression of FOXF2 in cervical cancer. High expression of FOXF2 inhibits the expression of target genes in the Wnt/β-catenin signalling pathway and β-catenin in the nucleus, thereby inhibiting the proliferation, invasion and migration of cervical cancer cells. Wang et al^34^. found that FOXF2 expression was decreased in OC tissues. The lncRNA ADAMTS9-AS2 could inhibit OC progression by inhibiting the expression of miR-182-5p to improve the expression of FOXF2^34^. Figueroa et al^70^. found that a SNP FOXF2rs1711973 variant was associated with a risk of bladder cancer in non-smokers, but the association was not found in people who smoke. Higashimori et al.^33^ confirmed that FOXF2 was preferentially methylated in GC and that its promoter methylation level, which could lead to the downregulation of FOXF2 expression, was higher than it was in normal gastric tissues. Furthermore, FOXF2 can inhibit Wnt signal transduction and the invasion and migration of GC cells by causing the ubiquitylation and degradation of β-catenin via inducing the expression of E3 ligase interferon regulatory factor 2-binding protein-like (IRF2BPL), and it can do so independent of glycogen synthase kinase-3β^33^. Moreover, FOXF2 can suppress the proliferation of GC cells by inhibiting the G1-S cell-cycle transition and by inducing apoptosis^33^. In intestinal adenomas of mice, FOXF2 can increase the expression of the Wnt inhibitor Secreted Frizzled Related Protein 1 (SFRP1) to repress Wnt signalling and thus negatively regulate the formation and growth of intestinal adenomas^40,41^. In an orthotopic RMS mouse model, the most common soft tissue sarcoma in children^71^, FOXF2 can transcriptionally inhibit p21 by binding to its promoter, consequently inducing the proliferation of cancer cells and promoting tumourigenesis of RMS^35^.

## Clinical significance of FOXF2 in tumours

FOXF2 plays an important role in tumours, but its role is not identical or even opposite in different tumours or different subtypes of the same tumour. Therefore, regulating the expression level of FOXF2 is an ideal treatment for tumours. Specifically, decreased FOXF2 expression is a marker of poor prognosis in grade II of TNBC^72^, NSCLC^20^, CRC^26^, ESCC^68^, GC^33^. Inhibitors of miR-200, miR-301, miR-182, miR-130, and miR-519 can be used to treat tumours. Targeting the FOXF2/BMP/SMAD axis may be a potential strategy for the treatment of bone metastasis of breast cancer^43^. In addition, FOXF2 may also be an ideal target for the treatment of RMS and HCC^32,35^.

The effects and mechanisms of FOXF2 in different tumours are summarized in Table 1.

## The association between FOXF2 and embryonic development

**Fig. 3:**
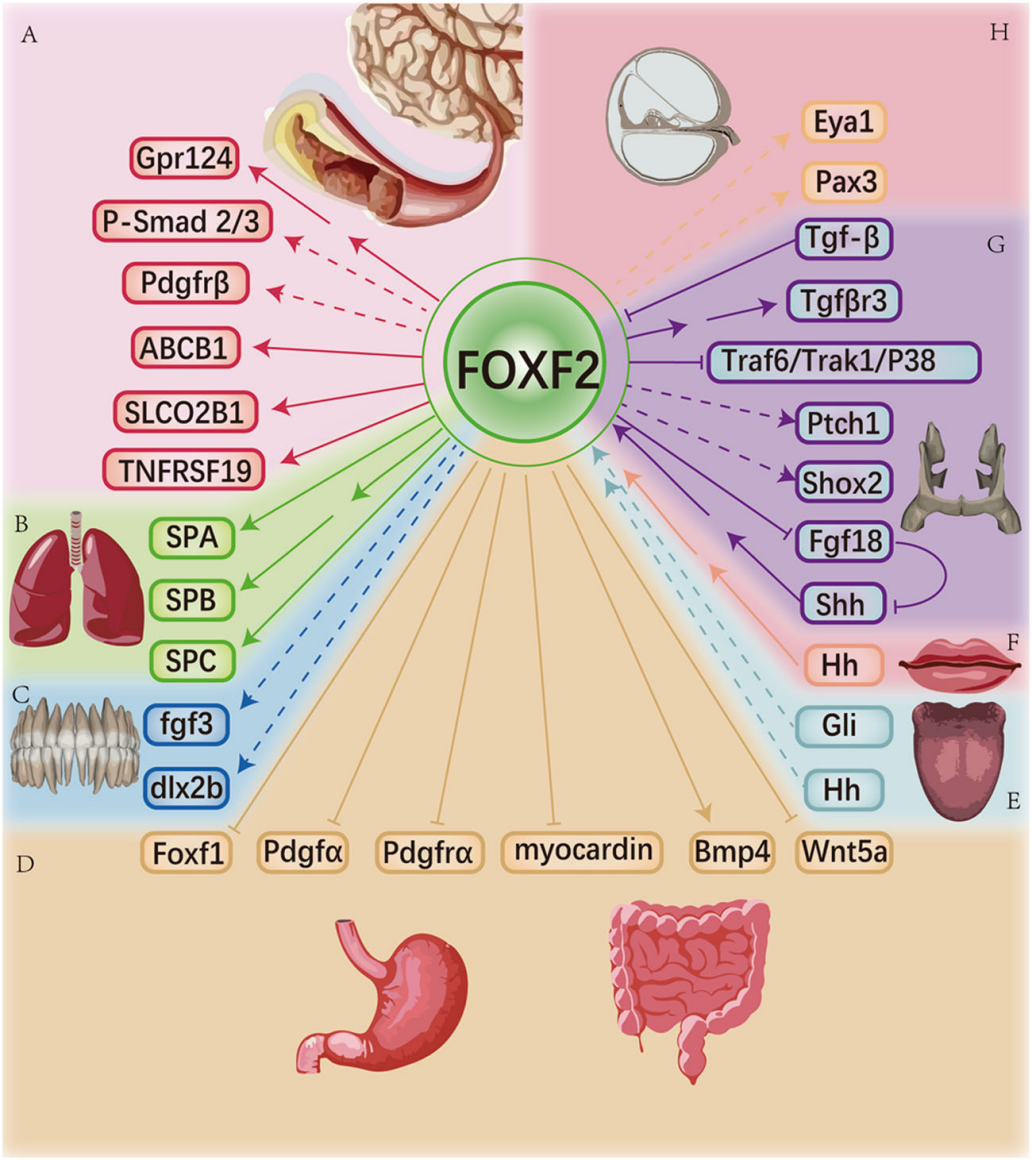
Genes upstream and downstream of FOXF2 in organs. **a** FOXF2 can upregulate the levels of Gpr124, P-Smad 2/3, Pdgfrβ, ABCB1, SLCO2B1, and TNFRSF19 during the development of blood vessels in the brain, which maintains the integrity of the BBB. **b** FOXF2 could induce the expression of some lung-specific genes to ensure lung function, i.e. SPA, SPB, and SPC. **c** FOXF2 modestly stimulates the expression of fgf3 and dlx2b, which prevent defects in tooth buds. **d** FOXF2 decreases the levels of Foxf1, Pdgfα, Pdgfrα, myocardin, and Wnt5a while stimulating Bmp4 expression, thus preventing excessive proliferation and metastasis of the gastrointestinal epithelium. **e** Hh and Gli tentatively stimulate FOXF2 expression in tongue development. **f** The Hh signal could indirectly stimulate FOXF2 expression, avoiding the occurrence of a cleft lip. **g** The Shh-FOXF2-Fgf18-Shh circuit controls the development of palate. At the same time, the levels of Ptch1 and Shox2 were attenuated in FOXF2 knockout mice. FOXF2 could indirectly stimulate Tgfβr3 expression and inhibit a Smad-independent TGF pathway namely, Traf6/Trak1/P38. **h** Activation of the TGF-β signalling pathway causes downregulation of FOXF2, which results in a cleft palate. FOXF2 tentatively stimulates Eya1 and Pax3 expression in developing cochlea. Protein names contain all uppercase letters for humans (e.g., ABCB1); all lowercase letters for zebrafish (e.g., fgf3); only the first letter capitalized for mice (e.g., Ptch1). ABCB1, ATP binding cassette subfamily B member 1; SLCO2B1, solute carrier organic anion transporter family member 2B1; TNFRSF19, TNF receptor superfamily member 19, dlx2b, distal-less homeobox 2b; Shox2, short stature homeobox 2; Ptch1, patched 1; and Pax3, paired box 3.

The FOXF2 protein is commonly found in animals^6^. Many studies have demonstrated that FOXF2 has vital effects on the embryonic development of the lip, tooth, tongue, eye, cochlea, palate, cerebrum, gastrointestinal (GI) tract, bronchus, lung, tendon, diaphragm, cartilage and heart^3,4,45–50,73–89^. The following is an overview of the association between FOXF2 and diseases mainly related to malformations that occur in these different tissue locations. The aberrant expression of FOXF2 and the mechanisms of corresponding diseases are summarized in Table 2. The upstream and downstream genes known to be associated with FOXF2 in different organs are summarized in Fig. 3.

**Table 2:** A summary of the aberrant expression of FOXF2 and the mechanism of related disease.

Expression location		Organ	Species	Related disease	Mechanism	Reference
Head	Face	Cochlea	Human	Sensorineural hearing loss	↓FOXF2	^3^
Head	Face	Cochlea	Mouse	Inner ear anomalies	↓Foxf2/↓Eya1, ↓Pax3	^3^
Head	Face	Lip	Mouse	Cleft lip	↓Hh signalling/↓Foxf2	^45^
Head	Face	Tongue	Mouse	Aglossia	Cilia mutation/↓GLIA/↓Foxf2	^46^
Head	Face	Eye	Human	Persistent hyperplastic primary vitreous	↑FOXF2	^49^
Head	Face	Eye	Human	Corectopia and dysplasia of anterior chamber of eyes	↓FOXF2	^76^
Head	Face	Tooth	Human	Teeth dysplasia	↓FOXF2	^74^
Head	Face	Palate	Mouse	Cleft palate	↓Foxf2/↓Tgfbr3, ↓integrins αV, ↓integrinsβ1/↓TGF-β signal	^80^
Head	Cranium	Brain	Mouse	Intracranial haemorrhage	↓Foxf2/↓TGF-β signal/↓Gpr124 mRNA	^4^
Digestive and respiratory systems	–	GI tract	Mouse	Excessive growth of intestinal epithelium	↓Foxf2/↑Wnt5a, ↓Bmp4	^47^
Digestive and respiratory systems	–	Lung	Human	Broncho-pulmonary dysplasia	↓FOXF2	^85^
Mesoderm-derived organs	–	Diaphragm	Human	Congenital diaphragmatic hernia	↓FOXF2	^88^
Mesoderm-derived organs	–	Cartilage	Zebrafish	Midline cartilage defects	↓foxf2/↓col2a1, ↓acan	^89^
Mesoderm-derived organs	–	Heart	Mouse	Atrioventricular septal defect	↓Foxf2	^50^

“↑” means upregulation, “↓” means downregulation; *Eya1* eyes absent 1, *Pax3* paired box 3, *Hh* hedgehog, *GLI* glucagonlike immunoreactivity, *Tgfbr3* transforming growth factor β receptor 3, *Gpr124* G protein-coupled receptor 124, *Wnt5a* wingless-type MMTV integration site family member 5a, *Bmp4* bone morphogenetic protein 4, *col2a1* collagen type II α1, *acan* aggrecan.

### In the development of head organs

#### Facial organs

During lip development, Shh signalling could directly target Foxf2 in mice, thus promoting the proliferation of the cranial neural crest during the formation of upper lip morphology and ensuring the normal development of the lip^45^. There is also a statistical association between two adjacent SNPs (rs732838, and rs1711968) in human *FOXF2* and non-syndromic cleft lip with or without cleft palate^73^. During tooth development, the deletion of *FOXF2* in humans could lead to tooth agenesis. In addition, the deletion may serve as a secondary cause giving rise to the facial malformations resulting from the hemizygous expression of *FOXC1*^74^. The mutation of foxf2 in zebrafish is associated with the loss of early tooth markers dlx2a and fgf3^89^. During tongue development, the mutation of cilia in neural crest cells is associated with the loss of GLIA activity and the inactivation of Foxf2, where it serves as the target gene of GLI in mice, resulting in aglossia^46^. During eye development, the mutation of *Foxf2* in mice could cause the deficiency of Schlemm’s canal, attenuation of the iris stroma and hyperplasia of the trabecular meshwork^75^. The low expression of human FOXF2 is also related to disease phenotypes, namely, corectopia and dysplasia of the anterior chamber of eyes^76^, whereas the overexpression of *FOXF2* in humans corresponds to persistent hyperplastic primary vitreous^49^. During cochlear development, FOXF2 in humans is highly associated with sensorineural hearing loss caused by cochlear dysfunction, and Foxf2 in mice maintains the expression of downstream genes related to cochlear development, such as Pax3 and Eya1^3^. During palate development, a missense mutation in human *FOXF2* may give rise to the vanished uvula^77^. In the developing murine palate mesenchyme, the expression of *Foxf2* is regulated by Shh-Smo signalling^78^, whereas Foxf2 in mice could sustain the expression of Shh in the palatal epithelium by suppressing the expression of *Fgf18* in the palatal mesenchyme^79^. In summary, the above processes constitute the Shh-Foxf-Fgf18-Shh circuit, which controls palatogenesis in embryos^79^. This study also shows that the expression of Ptch1 and Shox2 could be influenced in the developing palatal shelves of Foxf2 mutant mouse embryos^79^. Moreover, the levels of extracellular proteins related to TGF-β, namely, Tgfbr3, a fibronectin splice-isoform, and integrins αV and β1, were diminished in *Foxf2* knockout mice. In addition, the downregulation of TGF-β corresponds to the reduction of the extracellular matrix and inhibited proliferation. Thus, the absence of Foxf2 contributes to the formation of cleft palate^80^. In Foxf2 mutant palatal shelves, the Tgfβ pathway mediated by Traf6/Trak1/p38, which is essential to the palatal shelf mesenchyme, could also be enhanced^80^.

#### Cranial organs

Homayounfar et al.^81^ found a significant difference in *FOXF2* expression between metopic intrasutural mesenchyme/frontal bones and sagittal intrasutural mesenchyme/parietal bones. Thus, FOXF2 could act as a molecular marker for distinguishing the origin of one skull compartment from another. In murine embryos, vascular smooth muscle cells of arteries and pericytes of capillaries in the brain are derived from neural crest cells expressing Foxf2. Inactivation of Foxf2 gives rise to an unstable blood-brain barrier (BBB) in adult mice, as well as poor differentiation and hyperplasia in pericytes of embryos. Furthermore, *Foxf2* knockout mice express a decreased level of *Gpr124* mRNA in the brain because of halted TGF-β signalling, leading to intracranial haemorrhaging. The mutation of Foxf2 would increase Smad2/3 and attenuate *Pdgfrβ* expression, which may cause the destruction of BBB^4^. Another study also showed that FOXF2 could induce the expression of BBB markers ABCB1, TNFRSF19, and SLCO2B1 in human brain microvascular endothelial cells^82^.

### In the development of digestive and respiratory organs

Foxf2 regulates intestinal development by modulating the PDGF, SRF and Hedgehog signalling pathways in murine GI smooth muscle cells^83^. *Foxf2* expression in the mesoderm mainly plays roles in distal GI tract development. Interestingly, Foxf2 could be activated in endodermal cells by culturing them in fibroblast-conditioned medium containing Wnt3A^84^. *Foxf2* in mice also alters the expression of GI paracrine signalling molecules, namely, Bmp4 and Wnt5a. Specifically, mutating *Foxf2* represses *Bmp4* expression and activates *Wnt5a* expression in the developing intestines, resulting in obstruction of apoptosis and hyperplasia in epithelial cells. Foxf2 participates in the synthesis of collagen in the GI extracellular matrix^47^. The expression of Foxf1, Pdgfα, Pdgfrα, and myocardin could increase in the colonic smooth muscle in Foxf2 knock-out mice^83^. During lung development, FOXF2 in humans can bind the promoters of lung-specific genes, including pulmonary *SPA*, *SPB*, and *SPC*, to alter their expression^48^. Meanwhile, the study also shows a connection between the low expression of *FOXF2* and bronchopulmonary dysplasia in infants^85^. Foxf2 in mice could be detected in the developing lung mesenchyme, while Foxp2, another pivotal molecule functioning in regulating lung gene expression, is found in the bronchial epithelium, indicating that Foxf2 plays a specific role in the development of the embryonic lung^86^.

### In the development of other mesoderm-derived organs

*Foxf2* is not expressed in developing limbs during the first 12.5 days of embryonic development in mice; further, at E13.5, it is expressed in the dorsal rather than ventral limb tendons. Thus, Foxf2 could be regarded as a biomarker of differentiated dorsal limb tendons^87^. Gene enrichment analysis also suggests that FOXF2 is involved in diaphragm development^88^. Xu et al.^89^ found that foxf2 in zebrafish participated in facial cartilage development. Midline cartilage defects are observed in *foxf1*; *foxf2a* double mutants, and the defects were exacerbated by a mutation of *foxf2b*. Since Sox9 can bind cartilage genes *col2a1* and *acan* to modulate the synthesis of cartilage, researchers deduced that foxf2 binds to the enhancers of cartilage genes, thus facilitating the binding and activation of Sox9 in zebrafish^89^. In embryonic mice, Foxf2 and Foxf1a are selectively expressed in the second cardiac field rather than the heart, which suggests that the formation of the atrioventricular septal is a downstream result of hedgehog signalling^50^.

## Discussion

According to the different types or subtypes of tumours, the roles of FOXF2 are not the same or even opposite. FOXF2 shows inhibitory effects in most tumours. In lung cancer (except for non-small cell lung cancer^20,21^) and RMS in mice^35^, FOXF2 mainly shows a promotive effect. In HCC^23^ and breast cancer^18,19^, the effect of FOXF2 can be both to inhibit and to promote. FOXF2 can affect the EMT process, G1-S cell cycle transition^33,39^, Wnt/β-catenin pathway^33,40–42^, BMP/SMAD pathway^43^ and VEGF-C/VEGFR-3 pathway^44^ to promote or suppress the development of tumours. Additionally, the expression of *FOXF2* is downregulated by multiple microRNAs and LSD1^29^, and it is upregulated by TGF-β^37^, MAZ^62^ and SP1^56^. These findings suggest that FOXF2 may serve as a new potential marker for the clinical diagnosis and treatment of tumours. Foxf2 also plays an important role in embryonic development, where it mainly functions in cell differentiation and controlled proliferation. The overexpression of FOXF2 is associated with the regulation of cell differentiation and metabolism, while low or no expression due to chromosomal loss of FOXF2 usually leads to an absence of organs or tissues. In embryonic development, its role in GI development is still controversial. Ormestad et al^47^. observed excessive proliferation of the GI epithelium in *FOXF2* knockout mice, resulting from the Wnt5a signalling pathway. Wang et al.^90^ observed that the morphology and function of the GI tract in *FOXF2* knockout mice were normal due to the compensatory effect of *FOXF1*. Although the signalling pathways by which FOXF2 plays a role in development are well understood, the downstream genes and associated signalling pathways of FOXF2 in the eyes, teeth, diaphragm, and second cardiac region are still unclear^49,50,74,76,88^.

The different roles of FOXF2 in tumours may be due to differences in the tumour microenvironment, organ heterogeneity, or the complex functions of FOXF2 itself. Its specific mechanism needs further study to clarify its different roles. Most studies on the embryonic development mechanism related to FOXF2 are conducted in mice. Thus, whether its expression pattern in mice is completely consistent with that of humans remains to be studied. Additionally, it is worth studying the clinical significance of FOXF2.
